# BC-Box Motif-Mediated Neuronal Differentiation of Somatic Stem Cells

**DOI:** 10.3390/ijms19020466

**Published:** 2018-02-05

**Authors:** Hiroshi Kanno, Yuqun Xu, Taykua Miyakawa, Atsuhiko Kubo, Tetsuhiro Higashida, Nahoko Baily Kobayashi, Tetsuhiko Yoshida, Masaru Tanokura

**Affiliations:** 1 Department of Neurosurgery, International University of Health and Welfare School of Medicine, Atami 413-0012, Japan; 2 Department of Neurosurgery, Yokohama City University School of Medicine, Yokohama 236-0004, Japan; kubstar9@gmail.com (A.K.); tets018@gmail.com (T.H.); 3 Department of Applied Biological Chemistry, Graduate School of Agricultural and Life Sciences, The University of Tokyo, Tokyo 113-8657, Japan; xuyuqunxuyuqun@gmail.com (Y.X.); atmiya@mail.ecc.u-tokyo.ac.jp (T.M.); amtanok@mail.ecc.u-tokyo.ac.jp (M.T.); 4 Keio Advanced Research Centers, Keio University, Tsukuba 300-2611, Japan; nahoko_kobayashi@mail.toagosei.co.jp (N.B.K.); tyoshida@dmb.med.keio.ac.jp (T.Y.)

**Keywords:** BC-box motif, BC-box protein, skin-derived precursors, neuronal differentiation

## Abstract

Von Hippel-Lindau tumor suppressor protein (pVHL) functions to induce neuronal differentiation of neural stem/progenitor cells (NSCs) and skin-derived precursors (SKPs). Here we identified a neuronal differentiation domain (NDD) in pVHL. Neuronal differentiation of SKPs was induced by intracellular delivery of a peptide composed of the amino-acid sequences encoded by the NDD. Neuronal differentiation mediated by the NDD was caused by the binding between it and elongin C followed by Janus kinase-2 (JAK2) ubiquitination of JAK2 and inhibition of the JAK2/the signal transducer and activator of transcription-3(STAT)3 pathway. The NDD in pVHL contained the BC-box motif ((A,P,S,T)LXXX (A,C) XXX(A,I,L,V)) corresponding to the binding site of elongin C. Therefore, we proposed that other BC-box proteins might also contain an NDD; and subsequently also identified in them an NDD containing the amino-acid sequence encoded by the BC-box motif in BC-box proteins. Furthermore, we showed that different NDD peptide-delivered cells differentiated into different kinds of neuron-like cells. That is, dopaminergic neuron-like cells, cholinergic neuron-like cells, GABAnergic neuron-like cells or rhodopsin-positive neuron-like cells were induced by different NDD peptides. These novel findings might contribute to the development of a new method for promoting neuronal differentiation and shed further light on the mechanism of neuronal differentiation of somatic stem cells.

## 1. Introduction

Previously, we demonstrated that von Hippel-Lindau tumor suppressor protein (pVHL) promotes the neuronal differentiation of neural stem/progenitor cells (NSCs) and skin-derived precursors (SKPs) and suggested a relationship between it and neuronal differentiation [1,2,3]. pVHL-derived peptide containing the BC-box motif ((A,P,S,T)LXXX (A,C) XXX(A,I,L,V)) [4] functions to promote neuronal differentiation in those cells: neural stem cells [5,6], bone marrow stromal cells [7] and skin-derived precursors (SKPs) [8,9]. However, the molecular mechanism of neuronal differentiation by pVHL or the peptide has not been clarified, and the ability of other sites except for the elongin BC site in pVHL to induce neuronal differentiation have still not been examined. Although some BC-box proteins that have homology with pVHL are reported to promote neuronal differentiation [10,11,12,13,14,15], it has never been shown whether a BC-box protein except for pVHL-derived BC-box motif peptide can promote the neuronal differentiation.

Here, we first identified the neuronal differentiation domain (NDD) in pVHL, and then suggested the neuronal differentiation mechanism by NDD. In addition, we determined the common motif in BC-box proteins [4] having homology with pVHL that showed the greatest ability to promote neuronal differentiation. Furthermore, we asked which neuron-like cells could be induced by NDDs derived from different BC-box proteins. Herein, we show that NDDs derived from different BC-box proteins induced cells to differentiate into different kinds of neuron-like cells.

## 2. Results

### 2.1. Identification of a Neuronal Differentiation Domain in pVHL

**Figure 1:**
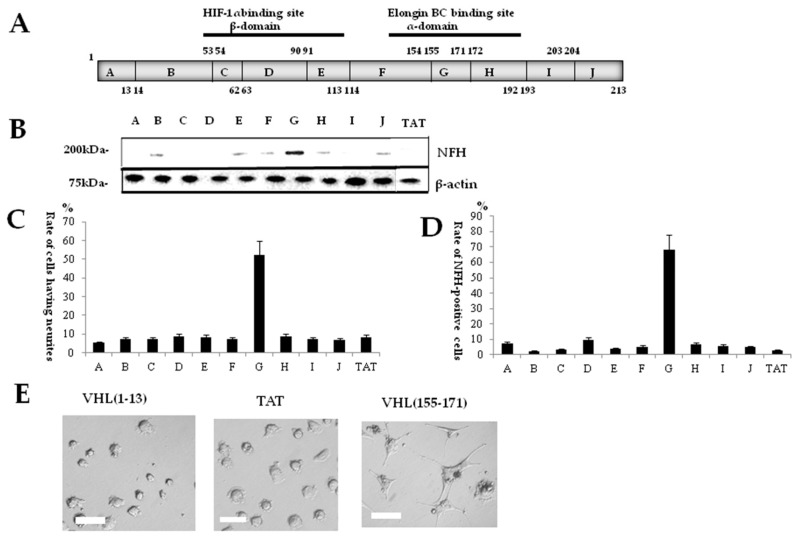
(**A**) Ten divided sequences of von Hippel-Lindau tumor suppressor protein (pVHL) and the relation to the structure of pVHL. The elongin BC binding site exists in α-domain; and the HIF-1α binding site, in β-domain; (**B**) Immunoblotting study with anti-Neurofilament-H (anti-NFH) antibody. The most potent expression of NFH was obtained with sequence G [VHL(155–171)]; (**C**) Immunocytochemical study with anti-NFH antibody. The greatest rate of immunoreactive cells for NFH was found at the “G” sequence (VHL(155–171)); (**D**) Rates of cells having neurites for the 10 divided sequences. The “G” sequence (VHL(155–171)) shows the greatest rate of cells having neurites. Phase-contrast microphotographs showed cells for “G” sequence (VHL(155–171)) and cells for “A” sequence [VHL(1–13)]; (**E**) The cells for “A” sequence (VHL(1–13)) and the cells for TAT showed no morphological change, as with non-treated cells (**left**), whereas the cells for “G” sequence [VHL(155–171)] assumed a neuron-like morphology (**right**). Scale bar = 20 μm.

To identify a neuronal differentiation domain (NDD), we divided pVHL into 10 functionally different sequence regions and then synthesized peptides conjugated with the protein transduction domain (PTD) corresponding to these 10 sequences (Figure 1A, Table 1). Neuronal differentiation of cells was evaluated in terms of protein expression assessed by Western blot analysis (Figure 1B), immunocytochemisty (Figure 1C), and neurite outgrowth activity (Figure 1D). The cells having neurites showed their greatest rate with VHL(155–171) (Figure 1D), and the immunocytochemical study on the cells showed the greatest rate of immunoreactivity at the VHL(155–171) in Neurofilament-H (NFH) (Figure 1C). Similarly, the Western blot study for NFH protein showed the greatest expression of this protein in the cells also with VHL(155–171) (Figure 1B). These results thus suggested that the VHL(155–171) sequence included a neuronal differentiation domain.

**Figure 2:**
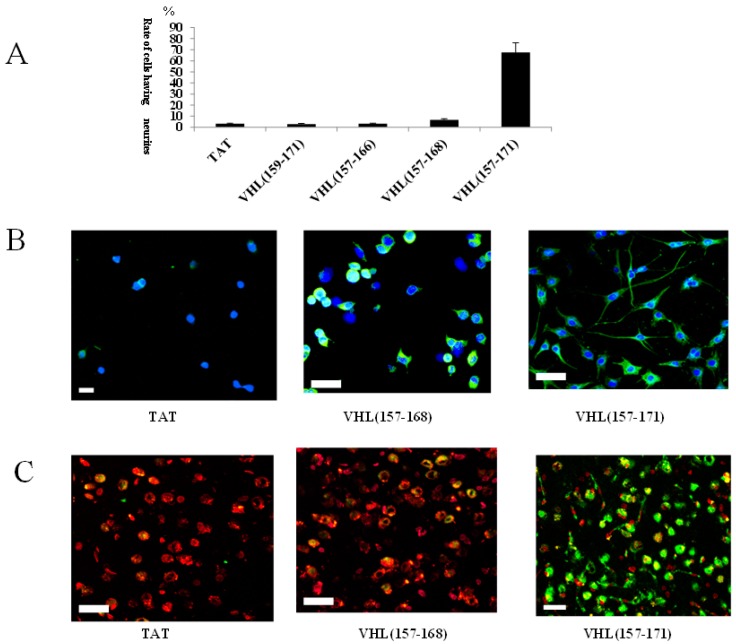
(**A**) Rates of cells having neurites for treated cells. The greatest of rate of cells having neurites was found for VHL(157–171) peptide-treated cells, with the rate being very low for the others; (**B**) Immunocytochemical microphotographs for TAT(YARAAARQARA)-treated cells (**left**), VHL(157–168)-treated cell (**center**), and VHL(157–171)-treated cells (**right**). Immunocytochemistry using anti-NFH antibody for neuron (green) and DAPI for nuclei (blue). Scale bar = 20 μm; (**C**) Confocal microscope images of engrafted cells with PKH26PCL-prelabeling in the non-treated group (**left**), VHL(157–168)-treated group (**center**), and the VHL(157–171)-treated group (**right**). Immunohistochemistry using anti-NeuN antibody (green) and PKH26PCL (red).

To identify this NDD in detail, we first examined the neurite outgrowth activities of TAT(YARAAARQARA), VHL(159–171), VHL(157–166), VHL(157–168), and VHL(157–171). As the results, VHL(157–171) showed neurite outgrowth activity to the same extent as VHL(155–171), whereas TAT, VHL(159–171), and VHL(157–166) scarcely did; VHL157–168 showed a significantly weaker neurite outgrowth activity than VHL(157–171) (*p* < 0.005, Figure 2A). In the immuocytochemical study using NFH, the percentage of NFH-positive cells was significantly higher in the VHL(157–171)-treated cells (60.4 ± 6.4%) than in the VHL(157–168)-treated cells, 10.1 ± 2.2%, *p* < 0.01; TAT[YARAAARQARA]-treated cells, 6.9 ± 1.5%, *p* < 0.005) (Figure 2B). In addition, immunohistochemical analysis revealed that VHL(157–171) peptide-treated cells differentiated to neuronal marker (NeuN)-positive cells in rat brain (positive rates of NeuN, 42.5 ± 4.5%), whereas VHL(157–168)-treated cells less differentiated to NeuN-positive cells (positive rates of NeuN, 9.2 ± 1.8%, *p* < 0.01) and TAT(YARAAARQARA)-treated cells scarcely differentiated (positive rates of NeuN, 3.2 ± 0.8%, *p* < 0.005) (Figure 2C).

**Figure 3:**
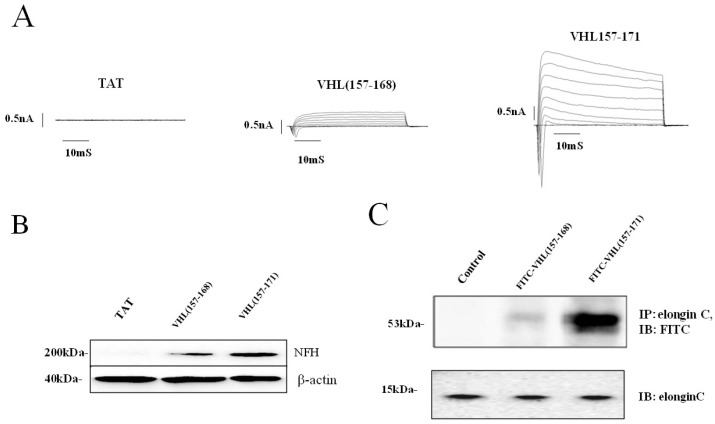
(**A**) Electrophysiological properties of peptide-treated cells. Voltage-gated inward and outward currents were recorded in the whole-cell patch-clamp configuration. (**Left**) TAT(YARAAARQARA)-treated cells. No currents are seen. (**Center**) VHL(157–168)-treated cells. Small outward K^+^ currents and fast inward Na^+^ currents are seen. (**Right**) VHL(157–171)-treated cells. Shown in the graph are large outward K^+^ currents and fast inward Na+ currents elicited by depolarizing voltage steps, which is a characteristic feature of a mature neuron; (**B**) Western blotting analysis using anti-NFH antibody for treated-treated cells. A distinct band for NFH was recognized for VHL(157–171)-treated cells, whereas a less distinct band for NFH for VHL(157–168)-treated cells (*p* < 0.01) and a faint band was found for TAT(YARAAARQARA)-treated cells (*p* < 0.001); (**C**) Immunoprecipitation (IP) with anti-elongin C using fluorescein-4-isothiocyanate (FITC)-VHL(157–171)-treated cells or FITC-VHL(157–168)-treated cells. A distinct band for FITC was recognized for FTIC-VHL(157–171)-treated cells, whereas a faint band for FITC was found for FITC-VHL(157–168)-treated cells and no band was found for non-treated cells.

Voltage-gated inward and outward currents were recorded in the whole-cell patch-clamp configuration. In whole-cell recordings of VHL(157–171) peptide-treated cells showing neurite outgrowth, the depolarizing voltage steps elicited both large outward potassium currents and fast inward Na^+^ currents, which are hallmark features of differentiated neurons. On the other hand, both significantly smaller outward potassium and inward Na^+^ currents were elicited in the whole-cell recording of VHL(157–168) peptide-treated cells than VHL(157–171) peptide-treated cells (*p* < 0.01) and no current was elicited in TAT(YARAAARQARA)-treated cells (Figure 3A).

In Western blotting analysis for cells three days after treatment, a significantly greater amount of anti-NFH protein was observed in the VHL(157–171)-treated cells than in the VHL(157–168)-treated cells (*p* < 0.01) or the TAT(YARAAARQARA)-treated cells (*p* < 000.1) (Figure 3B). On the other hand, the immunoprecipitation study revealed that FITC-conjugated VHL(157–171) peptide distinctly bound to elongin C but that FITC-conjugated VHL(157–168) significantly less did (*p* < 0.001, Figure 3C).

**Figure 4:**
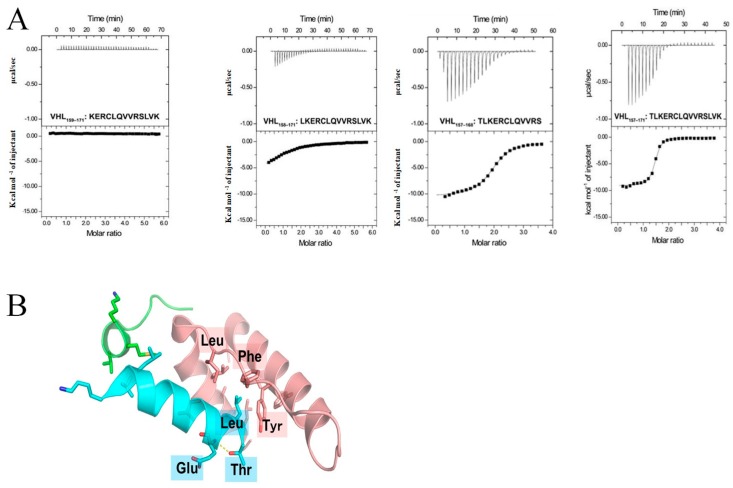
(**A**) Isothemal titration caloritetry (ITC) thermograms of elongin BC with various kinds of peptides. Results of ITC experiments by titrating elongin BC with VHL peptides are shown. The panels represent raw ITC data. Sequences of peptides used are shown in the corresponding thermograms. (**B**) Structure of elongin BC and VHL complex (PDB ID 4WQO). Elongin B, elongin C, and VHL are colored green, pink, and cyan, respectively. N-terminal residues of the BC-box of VHL are highlighted as cyan sticks and some relevant residues of elongin C are shown by pink sticks. Blue, red, and yellow represent nitrogen, oxygen, and sulfur atoms, respectively.

According to the results of the ITC experiments (Figure 4), the VHL(157–171) peptide bound to elongin BC with a dissociation constant (*K*_D_) of 0.54 ± 0.27 μM. One residue deleted from the N-terminus of the VHL(157–171) peptide resulted in more than a 30-fold loss of binding affinity for elongin BC (*p* < 0.001), and deletion of two residues from the N-terminus (VHL(159–171)) led to the complete loss of elongin BC binding affinity (*p* < 0.0001, Table 2). These results indicated that the N-terminal residues Thr and Leu were rather crucial for elongin BC binding, being consistent with the findings in an earlier study [16]. In contrast, deletion of three residues from the C-terminus of the VHL(157–171) peptide (VHL(157–168)) resulted in only brought an approximate four-fold loss of elongin BC binding ability (*p* < 0.01), indicating that there was less contact between the C-terminus of the VHL(157–171) peptide and elongin BC. Based on the crystal structure of the VHL-elongin BC complex, the N-terminal Thr residue of the VHL(157–171) peptide formed an intramolecular hydrogen bond with the Glu residue. Therefore, deletion of the Thr residue might have led to disruption of the helix formation at the N-terminus. The Leu residue of the VHL(157–171) peptide was crucial for elongin BC binding because of hydrophobic interaction with elongin C (Figure 4).

From these results, VHL(157–171) corresponding to BC-box motif ((A,P,S,T)LXXX (A,C) XXX(A,I,L,V)) and additional five amino acids on carboxyl terminal direction was identified as an NDD in pVHL.

### 2.2. JAK2 Ubiquitination and Neuronal Differentiation

**Figure 5:**
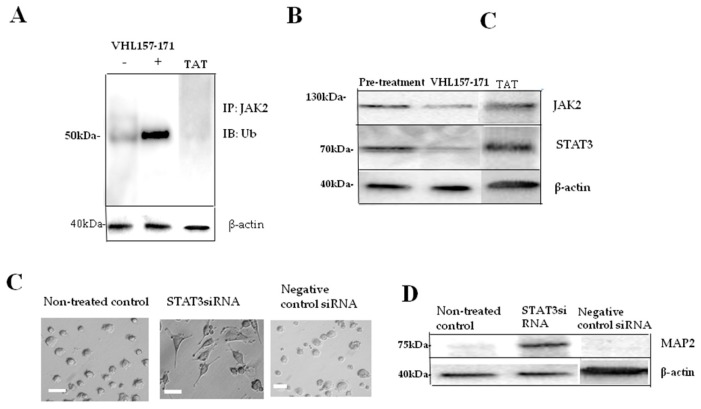
(**A**) Ubiquitination assay. IP with anti-JAK2 followed by immunoblotting (IB) with anti-ubiquitin. VHL(157–171) peptide was added (+) (**center lane**), not (−) (**left lane**), or TAT (**right lane**). When VHL(157–171) peptide was added, ubiquitination was significantly enhanced (*p* < 0.001); (**B**) Western blotting analysis using anti-JAK2 and anti-STAT3 for cells before and after treatment with VHL(157–171) peptide or TAT alone. Bands for JAK2 and STAT3 were found for pre-treated cells or TAT-treated cells more than post-treated cells (*p* < 0.01); (**C**) Morphological study on STAT3 siRNA-treated cells, negative control siRNA-treated cells, and non-treated cells. STAT3 siRNA-treated cells had significantly more neurites than negative control siRNA-treated cells (*p* < 0.01) or non-treated cells (*p* < 0.01). Scale bar = 20 μm; (**D**) Western blotting analysis using anti-MAP2 antibody for STAT3 siRNA-treated cells, negative control siRNA-treated cells, and non-treated cells. A significantly more distinct band was found for STAT3 siRNA-treated cells than negative control siRNA-treated cells (*p* < 0.01) or non-treated cells (*p* < 0.01).

The VHL protein belongs to the family of BC-box proteins which includes suppressor of cytokine signaling (SOCS) proteins. Since it is reported that SOCS proteins mediate Janus kinase-2 (JAK2) ubiquitination [17,18,19,20], we asked whether VHL(157–171) peptide-mediated neuronal differentiation was related to JAK2 ubiquitination or not. Thereby, we found the compound that contained VHL(157–171) peptide had activity as an E3 ubiquitin ligase and mediated JAK2 ubiqutination (Figure 5A). This result suggested VHL(157–171) peptide-mediated neuronal differentiation to be related to JAK2 ubiquitination. To confirm the following reaction, we asked whether JAK2/STAT3 was degraded or not. By Western blot analysis, we showed that JAK2 and STAT3 were indeed degraded (Figure 5B). Furthermore, we demonstrated that STAT3 siRNA-treated cells showed neurite outgrowth as observed by phase-contrast microscopy (Figure 5C) and expressed MAP-2 as shown by Western blotting analysis (Figure 5D).

### 2.3. Neuronal Differentiation Domains in Other BC-Box Proteins

Since the NDD in pVHL was identified as a BC-box motif plus five amino acids from the results, we next asked if NDDs in other BC-box proteins could be identified in the same manner as pVHL. The other BC-box proteins examined included suppressor of cytokine signaling-1 to 7 (SOCS1 to 7), ankyrin repeat and suppressor of cytokine signaling box-3 (ASB3), WD-40 repeats containing SOCS-box-2 (WSB2), and leucine-rich repeat protein-1 (LRR-1). So, BC-box motifs with five amino acids added in the carboxyl terminal direction were proposed as NDDs. In contrast, we also asked if amino-acid sequences composed of BC-box motif alone would have neuronal differentiation activity. First, after the addition of a peptide, the level of neurite outgrowth was evaluated. As a result, all BC-box protein-derived BC-box motif peptides with the additional five amino acids showed neurite outgrowth activities, though the level of neurite outgrowth was different for each. On the other hand, two of the peptides examined (derived from SOCS5 and SOCS7) composed of the BC-box motif only showed weak neurite outgrowth, and the remaining nine peptides did not show any neurite outgrowth (Table 3).

### 2.4. Protein Expression in BC-Box Protein-Derived Neuronal-Differentiation-Domain-Peptide-Mediated Neuronal Differentiation

**Figure 6:**
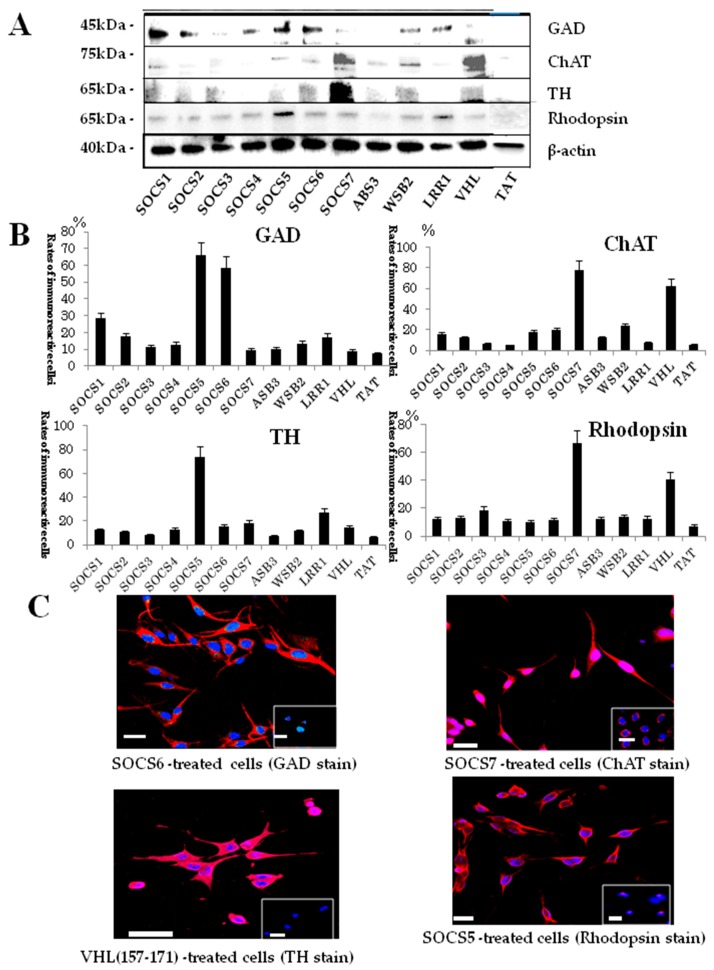
(**A**) Western blotting analyses of cells delivered neuronal-differentiation-domain (NDD) peptides for glutaminc acid decarboxylase (GAD), choline acetyltransferase (ChAT), tyrosine hydroxylase (TH), and rhodopsin; (**B**) Rates of immunoreactive cells treated by NDD peptides for ChAT, rhodopsin, TH, and GAD; (**C**) Characteristic immunocytochemical images of cells treated by NDD peptides for GAD, ChAT, TH, and rhodopsin. Upper left, SOCS6 peptide-treated cells stained by GAD. Upper right, SOCS7 peptide-treated cells stained by ChAT. Lower left, VHL(157–171) peptide-treated cells stained by TH. Lower right, SOCS5 peptide-treated cells stained by rhodopsin. GAD, ChAT, TH, and rhodopsin, expressions are indicated by TRIC (red). Nuclei, DAPI (blue). Small panels at lower right corners in all panels show images of negative control cells. Scale bars = 20 μm.

Protein expression levels in various NDD peptide-mediated neuronal differentiation were also examined by Western blot analysis, with the following results: Expression of TH was pronounced in SOCS7 peptide-treated cells, and moderate in SOCS 1, 3, 6, WSB2, and VHL(157–171)-treated cells. GAD was expressed highly in SOCS2, SOCS4, WSB2, and LRR1 peptide-treated cells. Expression of ChAT was high in SCOS7 and VHL(157–171) peptide-treated cells, and moderate in SOCS1 and WSB2 peptide-treated cells. Rhodopsin was highly expressed in SOCS5 peptide-treated cells, and moderate in SOCS2- and SOCS4 peptide-treated cells (Figure 6A). Finally, we immunocytochemically studied NDD-treated cells and determined the positive rates for the following neuronal markers: Glutamic acid decarboxylase (GAD, GABAnergic neuron marker), choline acetyltransferase (ChAT, cholinergic neuron marker), tyrosine hydroxylase (TH, dopaminergic neuron marker), and rhodopsin (retinal color epithelial cell marker). As a result, different cells delivered NDD peptides differentiated into different kinds of neuron-like cells. Briefly, high rates of positive cells were found for GABAnergic neuron-like cells treated by peptides derived from SOCS3-, ASN3-, WSM2-, LRR1-, and VHL; and for cholinergic neuron-like cells treated by a peptide derived from SOCS7. In addition, high rates of positive cells were found for TH-positive cells (dopaminergic neuron-like cells) treated by peptides derived from SOCS 1–3, 5, 6, ASB3, WSB2, LRR1, and VHL, and for rhodopsin-positive cells by peptides derived from SOCS5 and VHL (Figure 6B). Characteristic images of GAD, ChAT, TH, and rhodopsin-positive cells are shown in Figure 6C.

## 3. Discussion

We here observed the greatest neuronal differentiation activity for VHL(155–171) containing the elongin BC binding site termed the BC-box motif in the morphological study, immunocytochemistry, and Western blotting analysis. Furthermore, we recognized that the BC-box motif plus five amino acids (VHL(157–171)) played a role as an NDD within pVHL but that the BC-box motif plus two amino acids (VHL(157–168)) scarcely had neuronal differentiation activity in the morphological study, immunocytochemistry, immunohistochemistry, electophysiological study, and Wesntern blotting analysis. Thus, VHL(157–171) was identified as the NDD in the VHL protein. In addition, the immunoprecipitation study showed a firm binding between elongin C and BC-box motif plus five amino acids (VHL(157–171)) and a very weak binding between elongin C and BC-box motif plus two amino acids (VHL(157–168)). These findings were also supported by ITC assay that showed a firm binding between VHL(157–171) and elongin C, and a very weak binding between them. Thus, it was suggested that binding power between elongin C and a peptide containing the BC-box motif in pVHL was correlated with neuronal differentiation. After the binding reaction between VHL(157–171) and elongin C, JAK2 was ubiquitinated, resulting in inhibition of JAK2/STAT3 pathway and induction of neuronal differentiation. Our present study is partially supported by a previous one showing that VHL binds JAK2 to promote ubiquitin-mediated degradation of pJAX2. [19]. To confirm this hypothesis for the induction of neuronal differentiation, we demonstrated by using STAT3 siRNA that inhibition of STAT3, which is a transcriptional factor in the downstream of JAK2, led to neuronal differentiation.

Thus, since the NDD in pVHL was identified as a BC-box motif plus five amino acids, we asked if NDDs in other BC-box proteins could be identified in the same manner as pVHL, and BC-box motifs with five added amino acids in other BC-box proteins were also identified as NDDs. In addition, interestingly, different NDD peptide-treated cells differentiated into different kinds of neuron-like cells, but the mechanism involved is unknown. Since NDDs composed of different amino-acid sequences or complexes composed of different NDD peptides, elongin BC, and cullin would have different target proteins in the downstream, different kinds of neuron-like cells might be induced by different NDD peptides. Previously, we demonstrated that SPKs differentiated into TH-positive neuron-like cells in vitro with intracellular delivery of VHL protein-derived peptide and that these transplanted cells functioned as dopaminergic neurons in the brain of Parkinson’s disease model rats [9]. In our present study, TH-positive dopaminergic neuron-like cells, ChAT-positive cholinergic neuron-like cells, GAD-positive GABAnergic neuron-like cells, and rhodopsin-positive neuron-like cells were induced by different BC-box protein-derived NDD peptides. It would be promising for neuroregenerative medicine that specific neurons could be induced to differentiate from somatic stem cells.

In conclusion, the BC-box motif plus five amino acids at its C-terminus in BC-box proteins was identified as an NDD playing an important role in neuronal differentiation from SKPs. It was suggested that this differentiation was caused by binding between an NDD peptide and elongin C followed by inhibition of the JAK2/STAT3 pathway. Further, SKPs treated with different NDD peptides differentiated into different kinds of neuron-like cells. These novel findings might contribute to the development of a method for promoting neuronal differentiation and to education regarding the mechanism of neuronal differentiation of somatic stem cells.

## 4. Experimental Section

### 4.1. Peptide Design and Synthesis

To identify the NDD within pVHL, we divided the full-length pVHL into 10 functionally different parts. Then we designed peptides composed of amino acid sequences corresponding to each of them (Figure 1, Table 1). To facilitate the intracellular entry of peptides, we employed the protein transduction domain (PTD)-mediated peptide delivery system, by which these peptides were conjugated with PTD which consisting of modified TAT(YARAAARQARA) [21]. In addition, fluorescein-4-isothiocyanate (FITC) tag was added for the immunoprecipitation study. After identification of the NDD in pVHL, peptides comprising the BC-box motif and 5 amino acids C-terminal or the BC-box motif sequence alone in BC-box proteins were designed and neurite outgrowth activity was evaluated (Table 1).

Peptide synthesis has been described previously [5]. Briefly, the oligopeptide without free amino- or carboxyl end was synthesized based on the Fmoc (9-fluorenylmethyloxycarbonyl group) strategy. A solid support of an Fmoc-protected super acid-labile polyethyleneglycol resin (Fmoc-NH-SAL PEG, Watanabe Chem., Japan) was used for the solid support synthesis. After removal of the Fmoc group by piperidine, an Fmoc L-amino acid (Watanabe Chem., Tokyo, Japan) activated by 3 molar amounts of *O*-(7-azabenzotriazol-1-yl) *N*,*N*,*N*′,*N*′-tetramethyluronium hexafluorophosphate (HATU) and 3 molar amounts of 1-hydroxy-7-azabenzotiazole (HOAt); and then 6 molar amounts of *N*,*N*-diisopropylethylamine (DIPEA) were coupled to the resin. The amino acids were sequentially coupled to the resin, and the unreacted amino terminus in each coupling step was capped with acetic anhydride. The fully elongated oligopeptide was further treated with acetic anhydride after removal of the Fmoc group. The synthesized oligopeptide was cleaved from the resin and deblocked with trifluoroacetic acid (TFA) mixed with *m*-cresol, 1,2-ethanediol, thioanisole, and trimethylsilyl bromide (TMSBr). The deblocked peptide was purified by reversed-phase HPLC using an acetonitrile gradient (Tosoh, Tokyo, Japan), and the molecular weight of the peptide was confirmed by MALDI-TOF mass spectrometry (Applied Biosystems, Golden, CO, USA). The oligopeptide concentration was determined by the UV absorbance of the tyrosine residue attached at the N-terminal end of the sequence.

### 4.2. Cell Culture and Neuronal Differentiation

Rodent skin-derived precursor cells (SKPs), which can differentiate to neurons, were used. SKPs were previously isolated from the back dermis of neonate Wistar rats by the authors [8]. These cells were cultured in growth medium composed of DMEM/F12 (1:1; Gibco, Grand Island, NY, USA) containing 2% B27 supplement (Gibco), 20 ng/mL epidermal growth factor (EGF; Upstate Biotechnology, Lake Placid, NY, USA), and 40 ng/mL bFGF (PeproTech EC Ltd., Rocky Hill, NJ, USA) in a humidified incubator at 37 °C with 5% CO_2_. The floating clusters of cultured cells were centrifuged and dissociated into a single-cell suspension by use of a Pasteur pipette with a flame-polished tip. These single cells were then re-suspended into at a concentration of 50,000 cells/mL for passage or experiments. Our previous study showed that the SKPs could differentiate into various types of cells including adipose cells, muscle cells, and neuronal cells (astrocytes, neurons) [8].

For neuronal differentiation, cells were dissociated into a single-cell suspension by use of a Pasteur pipette and re-suspended into a concentration of 50,000 cells/mL in DMEM/F12 without serum, neurotrophic factors or growth factors. Then, the cells were cultured on poly-l-lysine-coated dishes or cover glasses, and the synthesized peptides at concentrations from 1 to 5 μM were added to the culture medium and delivered into the cells. Three days or more later, the cells were used for morphological, immunocytochemical, immunohistochemical, electrophysiological and immunoprecipitation studies, as well as for Western blot analysis.

### 4.3. Gene Knockdown

For gene silencing, STAT3 siRNA (Santa Cruz, San Diego, CA, USA) were used along with Transfection Reagent (101Bio, Palo Alto, CA, USA) according to manufacturer’s instructions.

### 4.4. Morphological Evaluation for Neuronal Differentiation

Neurite outgrowth with length exceeding the diameter of the neuronal soma is often assessed as a morphological index of neuronal differentiation. After a single-cell suspension was made by pipetting, the cells were cultured in DMEN/F12 medium alone at 37 °C in a 5% CO_2_ incubator. Thereafter, neurite outgrowth was evaluated with the following 4 levels: +++, for above 50% of the cells showing neurite outgrowth; ++, for 20–50% with neurite outgrowth; +, for 5–19% having neurite outgrowth; and −, for below 5% of the cells with neurite outgrowth.

### 4.5. Immunocytochemisty

Cultured cells were fixed with 4% paraformaldehyde in PBS (Phosphate buffered saline) for 10 min at room temperature. Cells were incubated for 1 h at room temperature with primary antibodies. Antibodies against anti-MAP2 antibody (Sigma-Aldrich, St. Louis, MO, USA), anti-Neurofilment-H (NFH) antibody (Sigma-Aldrich), anti-NeuN antibody (Merk Millipore, Billerica, MA, USA), anti-GFAP antibody (DAKO, Santa Clara, CA, USA), anti-tyrosine hydroxylase (TH) antibody (Merk Millipore, Billerica, MA, USA), anti-choline acetyl transferase (ChAT) antibody (Abbiotec, San Diego, CA, USA), anti-glutamic acid decaboxylase (GAD) antibody (Enzo Life Sciences, Flamingdale, NY, USA), and anti-rhodopsin antibody (Santa Cruz, San Diego, CA, USA) were used. After incubation with primary antibody, the cells were incubated for 1 h with secondary antibody, either rhodamine-conjugated anti-mouse IgG (Sigma-Aldrich) or FITC-conjugated anti-rabbit IgG (Sigma-Aldrich). Nuclear counterstaining was done with DAPI (Molecular Probes, Eugene, OR, USA) or TO-PRO-3 (Molecular Probes), and observations were made with a fluorescence microscope system (FV300, Olympus, Tokyo, Japan). We counted the number of immuno-positive cells with a given antibody in 4 to 6 random non-overlapping visual fields (50–200 cells per field) in each experiment. At least 2 experiments were performed per condition. The degree of positivity was expressed as the rate of immuno-positive cells to the total number of nuclei stained with DAP1 or TO-PRO-3.

### 4.6. Immunohistochemistry

To evaluate neuronal differentiation of transplanted cells in rat brains, immunohistochemical study was performed as described previously [1]. Before grafting, cells were preincubated with red fluorescence PKH26PCL (Sigma, St. Louis, MO, USA). Transplanted cells were divided to three groups: VHL(157–171) peptide-treated cells; VHL(157–168) peptide-treated cells; and TAT-treated cells. Rats were anesthetized with isoflurane before surgery, and 1 × 10^5^ cells were transplanted into rat brain. The rats were perfused with periodate-lysine-paraformaldehyde solution 6 weeks after the transplantation. Their brains were subsequently dissected and postfixed in the same medium for 2 h, cryopreserved in 30% sucrose for 12 h, and then embedded in Tissue Tek OCT compound (Sakura, Tokyo, Japan). Cryostat coronal sections of 14-μm thickness were prepared for immunohistochemistry. For immunostaining, sections were incubated with primary antibody, anti-NeuN antibody, and then incubated in secondary antibody, FITC-conjugated anti-rabbit IgG. Counterstaining was done with DAPI. In immohistochemistry, observations were made with a confocal immunofluorescence microscope (FV300, Olympus, Tokyo, Japan).

### 4.7. Western Blotting

Cultured cells were washed 3 times in cold PBS and then scraped into ice-cold PBS. After incubation on ice for 10 min, the cells were lysed with lysis buffer and centrifuged, after which the supernatants were collected. Each sample was separated by SDS-PAGE under reducing conditions and transferred electrophoretically to nitrocellulose filters. Non-specific binding of antibody was blocked by incubation with 5% donkey serum for 1 h. Western blots were probed with anti-MAP2 antibody, anti-NFH antibody, anti-ChAT antibody, anti-GAD antibody, anti-rhodopsin antibody, anti-TH antibody, and anti-elongin C antibody (Santa Cruz, San Diego, CA, USA) followed by horseradish peroxidase-conjugated secondary antibodies. Protein bands were detected by using a chemical luminescence detection system (ECL Plus Western Blotting Reagent Pack, Amersham, Hemel Hempstead, UK). Images were analyzed with LAS-1000 (Fujifilm, Tokyo, Japan), and the density of the bands was determined by using Image Gauge software (Fujifilm).

### 4.8. Immunoprecipitation

Cultured cells (1 × 10^6^) were washed with ice-cold PBS and lysed in RIPA lysis buffer (Upstate, Charlottesville, VA, USA). The total protein was extracted from the cells, and the total protein concentration of each lysate was adjusted to 1 mg/mL. The lysates were immunoprecipitated with anti- elongin C antibody (Santa Cruz, San Diego, CA, USA) diluted at 1:500 by using μMACS Protein A Microbeads (Miltenyi Biotec, Bergisch Gladbach, Germany). The immunoprecipitates were analyzed by SDS/gradient polyacrylamide gel and Western blotting with anti-FITC antibody (Santa Cruz, San Diego, CA, USA) diluted 1:500. Immunolabeled bands were detected by using enhanced chemiluminescence reagents (Amersham Biosciences Corp., Piscataway, NJ, USA). Images were analyzed with LAS-1000 (Fujifilm), and the density of the bands was determined by using Image Gauge software (Fujifilm).

### 4.9. Electrophysiology

To record fast sodium and delayed rectifier potassium currents, we prepared extracellular and intracellular solutions as described previously [3,5]. Five days after the addition of VHL(157–171) peptide at a 3-μM concentration or the addition of VHL157–168 peptide at the same concentration, a holding potential of −80 mV and voltage step of 20 mV over the range of −100 to 100 mV with 50 ms durations were applied to the recorded cells through patch electrodes. For recordings and data analysis we used CEZ-2300 (Nihon Kohden, Tokyo, Japan) and pCLAMP 6.0 software (Axon Instruments, Burlingame, CA, USA). Linear components of leak and capacitive currents were reduced by analogue circuitry and then canceled by the P/N method. Signals were sampled every 20 μs, and currents were filtered at 5 kHz. Data were additionally processed with Origin 5.0 (Microcal, Northhampton, MA, USA).

### 4.10. Cloning, Overexpression and Purification of Recombinant Proteins for Isothermal Titration Calorimetry

In order to determine the crucial peptide sequence for elongin BC binding by isothemal titration caloritetry (ITC), recombinant proteins of elongin B and elongin C were prepared as follows. The cDNA encoding elongin B and elongin C were respectively amplified by one-step reverse transcript PCR from human liver total RNA by using a PrimeScript One Step RT-PCR kit Ver.2 (Takara Bio Inc., Kusatsu, Japan). RT-PCR primers designed for elongin B (residues 1-118) were 5′-CGGGATCCGATGGACGTGTTCCTCATG-3′ (forward) and 5′-CCCAAGCTTTTATCACTGCACGGCTTG-3′ (reverse); and those for elongin C (residues 17–112) [22], 5′-GGAAGATCTGTATGTCAAATTGATATCATCTG-3′ (forward) and 5′-CCGCTCGAGTCATTAACAATCTAAGAAG-3′ (reverse). The amplified DNA fragments of elongin B and elongin C were cloned into the pCDFDuet-1 vector (Novagen, Madison, WC, USA) and used to transform *E. coli* strain BL21 (DE3) (Novagen). Overexpression was performed after the OD_600_ of the cell culture had reached 0.6 and was induced by incubation with 0.5 mM IPTG overnight at 25 °C. The cells were harvested by centrifugation at 5000 rpm for 10 min and stored at −80 °C before use. For purification, cell pellets were lysed by sonication in buffer A (20 mM Tris-HCl, pH 8.0, 0.5 M NaCl, 10 mM imidazole), and the soluble fraction was loaded onto Ni-NTA resin (Qiagen, Venlo, Netherlands) and eluted with buffer B (20 mM Tris-HCl (pH 8.0), 0.5 M NaCl, 250 mM imidazole). Eluted proteins were then loaded onto a Mono Q column followed by a Superdex 75 10/300 GL column (GE Healthcare, Chicago, IL, USA). For the ITC experiments, the elongin BC solution was exchanged with buffer C (20 mM HEPES-NaOH (pH 8.0), 150 mM NaCl) by using size-exclusion chromatography.

### 4.11. Isothermal Titration Calorimetry

Assays to assess the binding of peptides to elongin BC were performed by using a MicroCal iTC_200_ isothermal titration calorimeter (GE Healthcare, Chicago, IL, USA). Four types of VHL peptides were designed as shown in Table 2 and were synthesized by CS Bio Co. (Menlo Park, CA, USA). The concentration of elongin BC was adjusted to a final one of 35 μM and injected into the sample cell (204 μL). One mM peptide was titrated into the protein solution as 40 consecutive 1.0-μL aliquots at 120-s intervals at 25 °C. The first injection volume was 0.4 μL, and the observed thermal peak was excluded from the data analyses. Duplicate experiments were performed independently. Data were fitted by using the “one set of sites” mode of Origin software. The *K*_D_ values were calculated from duplicate thermograms.

### 4.12. Ubiquitination Assay

We asked whether VHL(157–171) peptide-mediated neuronal differentiation was related to ubiquitination or not. Then, we examined whether or not the delivery of VHL(157–171) peptide would promote the ubiquitination of JAK2. The ubiquitination reaction was carried out with the addition of 10 mM ubiquitin (Sigma-Aldrich), 2 mM Ubiquitin Activating Enzyme Solution (E1; Enzo Life Sciences, Inc., Farmingdale, NY, USA), 2 mM Ubiquitin Conjugating Enzyme Solution (E2; Enzo Life Sciences, Inc.), and VHL(157–171) peptide or no peptide. After incubation for 4 h, cultured cells (1 × 10^6^) were washed with ice-cold PBS and lysed in RIPA lysis buffer (Upstate, Charlottesville, VA, USA). The total protein was extracted from the cells, and the lysates were immunoprecipitated with anti-JAK2 antibody (Santa Cruz, San Diego, CA, USA) by using Protein A/G Sepharose (abcam, Cambridge, MA, USA). The immunoprecipitates were supplemented with 1 mM PMSF and the protease inhibitor cocktail. After 2 h, each sample was separated by SDS-PAGE and transferred electrophoretically to nitrocellulose filters. Western blots were probed with anti-ubiquitin antibody (Sigma-Aldrich) followed by horseradish peroxidase-conjugated secondary antibodies (Amersham). Protein bands were detected by using a chemical luminescence detection system (Amersham). Images were analyzed with LAS-1000 (Fujifilm).

### 4.13. Approval of Animal Experiment

Wistar rats (Clea, Tokyo, Japan) were used in all experiments and housed in a temperature controlled room on a 12-h day/night cycle with free access to food and water. All animal experimental procedures were approved by the Institutional Animal Use Committee of Yokohama City University (ID 153, 1 April 2015) and were in accordance with National Institutes of Health guidelines for care and use of laboratory animals.

### 4.14. Statistical Analysis

All numerical data were expressed as mean ± SEM. The rate of immunoreactive cells was determined as the percentage of positively stained cells in a random sample of 500 cells. Factorial analysis of variance (ANOVA) was applied to each group with pairwise comparison done by the Bonferroni method. Statistical significance was set at *p* < 0.05.

## Figures and Tables

**Table 1:** List of synthetic peptides.

Peptide Name	Amino Acid Sequence
TAT	YARAAARQARA
VHL(1–13)	YARAAARQARAMPRRAENWDEAEV
VHL(14–53)	YARAAARQARAGAEEAGVEEYGPEEDGGEESGAEESGPEESGPEELGAEEE
VHL(54–62)	YARAAARQARAMEAGRPRPV
VHL(63–90)	YARAAARQARALRSVNSRE PSQVIFCNRS PRVVLPVWLN
VHL(91–113)	YARAAARQARAFDGEPQPYPTLPPGTGRRIHSYR
VHL(114–154)	YARAAARQARAGHLWLFRDAGTHDGLV NQTELFVPSLNVDG
VHL(155–171)	YARAAARQARAVYTLKERCLQVVRSLVK
VHL(172–192)	YARAAARQARAPENYRRLDIVRSLYEDLEDHP
VHL(193–203)	YARAAARQARANVQKDLERLTD
VHL(204–213)	YARAAARQARAERIAHQRMGD
VHL(159–171)	YARAAARQARAKERCLQVVRS
VHL(157–166)	YARAAARQARA TLKERCLQVV
VHL(157–168)	YARAAARQARATLKERCLQVVRS
VHL(157–171)	YARAAARQARATLKERCLQVVRSLVK
FITC-VHL(157–168)	YARAAARQARATLKERCLQVVRS
FITC-VHL(157–171)	YARAAARQARATLKERCLQVVRSLVK
SOCS1-A	YARAAARQARA PLQELCRQRI
SOCS1-B	YARAAARQARAPLQELCRQRIVAAVG
SOCS2-A	YARAAARQARATLQHFCRLAI
SOCS2-B	YARAAARQARATLQHFCRLAINKCT G
SOCS3-A	YARAAARQARATLQHLCRKTV
SOCS3-B	YARAAARQARATLQHLCRKTVNGHLD
SOCS4-A	YARAAARQARASLQHICRTVI
SOCS4-B	YARAAARQARASLQHICRTVICNCTT
SOCS5-A	YARAAARQARA SLQYICRAVI
SOCS5-B	YARAAARQARASLQYICRAVICRCTT
SOCS6-A	YARAAARQARASLQYLCRFVI
SOCS6-B	YARAAARQARASLQYLCRFVIRQYTR
SOCS7-A	YARAAARQARASLQHLCRFRI
SOCS7-B	YARAAARQARASLQHLCRFRIRQLVR
ASB3-A	YARAAARQARASLTHLCRLEI
ASB3-B	YARAAARQARASLTHLCRLEIRSSIK
WSB2-A	YARAAARQARA SLKHLCRKAL
WSB2-B	YARAAARQARASLKHLCRKALRSFLT
LRR1-A	YARAAARQARATLLESSARTI
LRR1-B	YARAAARQARATLLESSARTILHNRI

Underline = protein transduction domain; double underline = BC box motif; all species = human.

**Table 2:** Equilibrium dissociation constants and thermodynamic parameters determined by ITC assays.

Name	Sequence	*K*_D_ (μM)	Δ*H* (kcal/mol)	Δ*S* (cal/mol/deg)	*N* (Sites)
VHL_157–171_	TLKERCLQVVRSLVK	0.54 ± 0.27	−7.99 ± 1.60	2.01 ± 4.30	1.64 ± 0.32
VHL_158–171_	LKERCLQVVRSLVK	18.7 ± 8.8	−7.82 ± 1.52	−4.38 ± 4.15	1.12 ± 0.01
VHL_159-171_	KERCLQVVRSLVK	-	-	-	-
VHL_157–168_	TLKERCLQVVRS	2.06 ± 0.09	−10.3 ± 0.2	−8.54 ± 0.74	1.89 ± 0.07

Data are shown as the mean ± standard deviation (S.D.) of two independent replicates.

**Table 3:** Neurite outgrowth activity of cells after BC-box protein-derived peptide delivery.

Derivation	Sequence	Neurite Outgrowth Activity	Sequence	Neurite Outgrowth Activity
SOCS-box family				
SOCS1	PLQELCRQRI	-	PLQELCRQRIVAAVG	++
SOCS2	TLQHFCRLAI	-	TLQHFCRLAINKCTG	+
SOCS3	TLQHLCRKTV	-	TLQHLCRKTVNGHLD	++
SOCS4	SLQHICRTVI	-	SLQHICRTVICNCTT	++
SOCS5	SLQYICRAVI	+	SLQYICRAVICRCTT	+++
SOCS6	SLQYLCRFVI	-	SLQYLCRFVIRQYTR	++
SOCS7	SLQHLCRFRI	+	SLQHLCRFRIRQLVR	+++
ASB3	SLTHLCRLEI	-	SLTHLCRLEIRSSIK	++
WSB2	SLKHLCRKAL	-	SLKHLCRKALRSFLT	++
VHL-box family				
hLRR-1	TLLESSARTI	-	TLLESSARTILHNRI	++
VHL	TLKERCLQVV	-	TLKERCLQVVRSLVK	+++

Underline = BC-box motif. +++, for above 50% of the cells showing neurite outgrowth; ++, for 20–50% with neurite outgrowth; +, for 5–19% having neurite outgrowth; and −, for below 5% of the cells with neurite outgrowth.
